# SPAK inhibitor ZT‐1a attenuates reactive astrogliosis and oligodendrocyte degeneration in a mouse model of vascular dementia

**DOI:** 10.1111/cns.14654

**Published:** 2024-03-03

**Authors:** Mohammad Iqbal H. Bhuiyan, Khadija Habib, Md Tipu Sultan, Fenghua Chen, Israt Jahan, Zhongfang Weng, Md Shamim Rahman, Rabia Islam, Lesley M. Foley, T. Kevin Hitchens, Xianming Deng, Scott W. Canna, Dandan Sun, Guodong Cao

**Affiliations:** ^1^ Department of Pharmaceutical Sciences, School of Pharmacy University of Texas at El Paso El Paso Texas USA; ^2^ Department of Neurology University of Pittsburgh Pittsburgh Pennsylvania USA; ^3^ Pittsburgh Institute for Neurodegenerative Disorders University of Pittsburgh Pittsburgh Pennsylvania USA; ^4^ Veterans Affairs Pittsburgh Health Care System Pittsburgh Healthcare System Geriatric Research Education and Clinical Center Pittsburgh Pennsylvania USA; ^5^ Nostrum Hospital Dhaka Bangladesh; ^6^ Animal Imaging Center University of Pittsburgh Pittsburgh Pennsylvania USA; ^7^ Department of Neurobiology University of Pittsburgh Pittsburgh Pennsylvania USA; ^8^ State Key Laboratory of Cellular Stress Biology, School of Life Sciences Xiamen University Xiamen Fujian China; ^9^ Department of Pediatric Rheumatology The Children's Hospital of Philadelphia Philadelphia Pennsylvania USA

**Keywords:** astrogliosis, BCAS, NKCC1, vascular dementia, VCID, ZT‐1a

## Abstract

**Background:**

Astrogliosis and white matter lesions (WML) are key characteristics of vascular contributions to cognitive impairment and dementia (VCID). However, the molecular mechanisms underlying VCID remain poorly understood. Stimulation of Na‐K‐Cl cotransport 1 (NKCC1) and its upstream kinases WNK (with no lysine) and SPAK (the STE20/SPS1‐related proline/alanine‐rich kinase) play a role in astrocytic intracellular Na^+^ overload, hypertrophy, and swelling. Therefore, in this study, we assessed the effect of SPAK inhibitor ZT‐1a on pathogenesis and cognitive function in a mouse model of VCID induced by bilateral carotid artery stenosis (BCAS).

**Methods:**

Following sham or BCAS surgery, mice were randomly assigned to receive either vehicle (DMSO) or SPAK inhibitor ZT‐1a treatment regimen (days 14–35 post‐surgery). Mice were then evaluated for cognitive functions by Morris water maze, WML by ex vivo MRI‐DTI analysis, and astrogliosis/demyelination by immunofluorescence and immunoblotting.

**Results:**

Compared to sham control mice, BCAS‐Veh mice exhibited chronic cerebral hypoperfusion and memory impairments, accompanied by significant MRI DTI‐detected WML and oligodendrocyte (OL) death. Increased activation of WNK‐SPAK‐NKCC1‐signaling proteins was detected in white matter tissues and in C3d^+^GFAP^+^ cytotoxic astrocytes but not in S100A10^+^GFAP^+^ homeostatic astrocytes in BCAS‐Veh mice. In contrast, ZT‐1a‐treated BCAS mice displayed reduced expression and phosphorylation of NKCC1, decreased astrogliosis, OL death, and WML, along with improved memory functions.

**Conclusion:**

BCAS‐induced upregulation of WNK‐SPAK‐NKCC1 signaling contributes to white matter‐reactive astrogliosis, OL death, and memory impairment. Pharmacological inhibition of the SPAK activity has therapeutic potential for alleviating pathogenesis and memory impairment in VCID.

## Graphical abstract

Cerebral hypoperfusion‐induced increased activity of the SPAK‐NKCC1 pathway contributes to reactive astrogliosis and subsequent demyelination and cognitive impairment in a mouse model of vascular dementia. SPAk inhibitor ZT‐1 improved cognitive impairment by preventing reactive astrogliosis and demyelination. Therefore, SPAK‐NKCC1 can be a potential therapeutic target for treating vascular dementia.

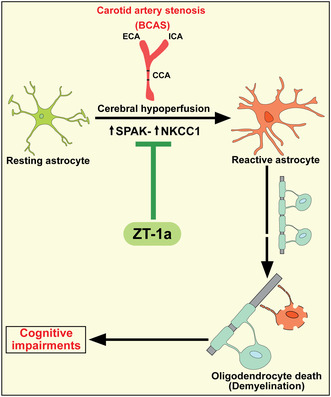

## 1 INTRODUCTION

Vascular contributions to cognitive impairment and dementia (VCID) are currently considered as one of the leading causes of dementing illness in the world and are emerging as a major public health problem in many countries.^1^, ^2^ Since there are no approved drugs for VCID treatment, the development of effective therapy is urgently required. Reactive astrogliosis, blood–brain barrier (BBB) disruption, and white matter lesion (WML) represent the pathological hallmark of VCID.^3^, ^4^, ^5^ The most common feature of clinical VCID is diffuse WML, detected as areas of hyperintensity on T2‐weighted magnetic resonance (MR) images.^2^, ^6^, ^7^ Chronic cerebral hypoperfusion (CCH), resulting from either small vessel disease or critical arterial stenosis (carotid artery atherosclerosis or arteriosclerosis), causes oligodendrocyte (OL) death, WML, and subsequent cognitive impairment. However, the underlying molecular and cellular mechanisms by which reactive astrogliosis and WML occur are not well understood.

Evolutionary conserved WNK (“with no lysine” [K]) kinases, along with the downstream SPAK/OSR1 (Ste20/SPS1‐related proline/alanine‐rich kinase and oxidative stress‐responsive kinase 1) kinases, regulate activities of multiple‐ion transporters and channels via protein phosphorylation.^8^, ^9^ One of the major targets of WNK and SPAK/OSR1 is Na^+^‐K^+^‐2Cl^−^ cotransporter isoform 1 (NKCC1),^10^ which has been implicated in the pathogenesis of multiple brain disorders.^11^ Interestingly, studies from our laboratory and others found that NKCC1 activity causes astrocytic intracellular Na^+^ overload, hypertrophy, and swelling and leads to astrogliosis after in vitro and in vivo ischemia or ammonia‐induced toxicity.^12^, ^13^, ^14^, ^15^ This highlights NKCC1 protein as a potential therapeutic target for various CNS diseases associated with reactive astrogliosis, including VCID. Therefore, in this study, we investigated the involvement of the WNK‐SPAK‐NKCC1 cascade in bilateral carotid artery stenosis (BCAS)‐induced reactive astrogliosis and WML. We also investigated the effect of the non‐ATP competitive specific inhibitor of SPAK kinase ZT‐1a in mitigating SPAK‐NKCC1‐mediated reactive astrogliosis, OL death, and memory impairment in a mouse BCAS model. Here, we report that CCH triggered stimulation of the WNK‐SPAK‐NKCC1 cascade in white matter astrocytes and led to reactive astrogliosis and demyelination. Most importantly, post‐BCAS administration of ZT‐1a effectively attenuated VCID pathologies and significantly improved learning and memory function in BCAS mice.

## 2 METHODS

### 2.1 Materials

Vendor and material information were included in the Appendix S1 and S2.

### 2.2 Animals and modified BCAS models

All animal studies were approved by the University of Pittsburgh and the University of Texas at El Paso Institutional Animal Care and Use Committee, which adhere to the National Institutes of Health Guide for the Care and Use of Laboratory Animals, and reported in accordance with the Animal Research: Reporting In Vivo Experiments (ARRIVE) guidelines.^16^ All efforts were made to minimize animal suffering and the number of animals used.

**FIGURE 1:**
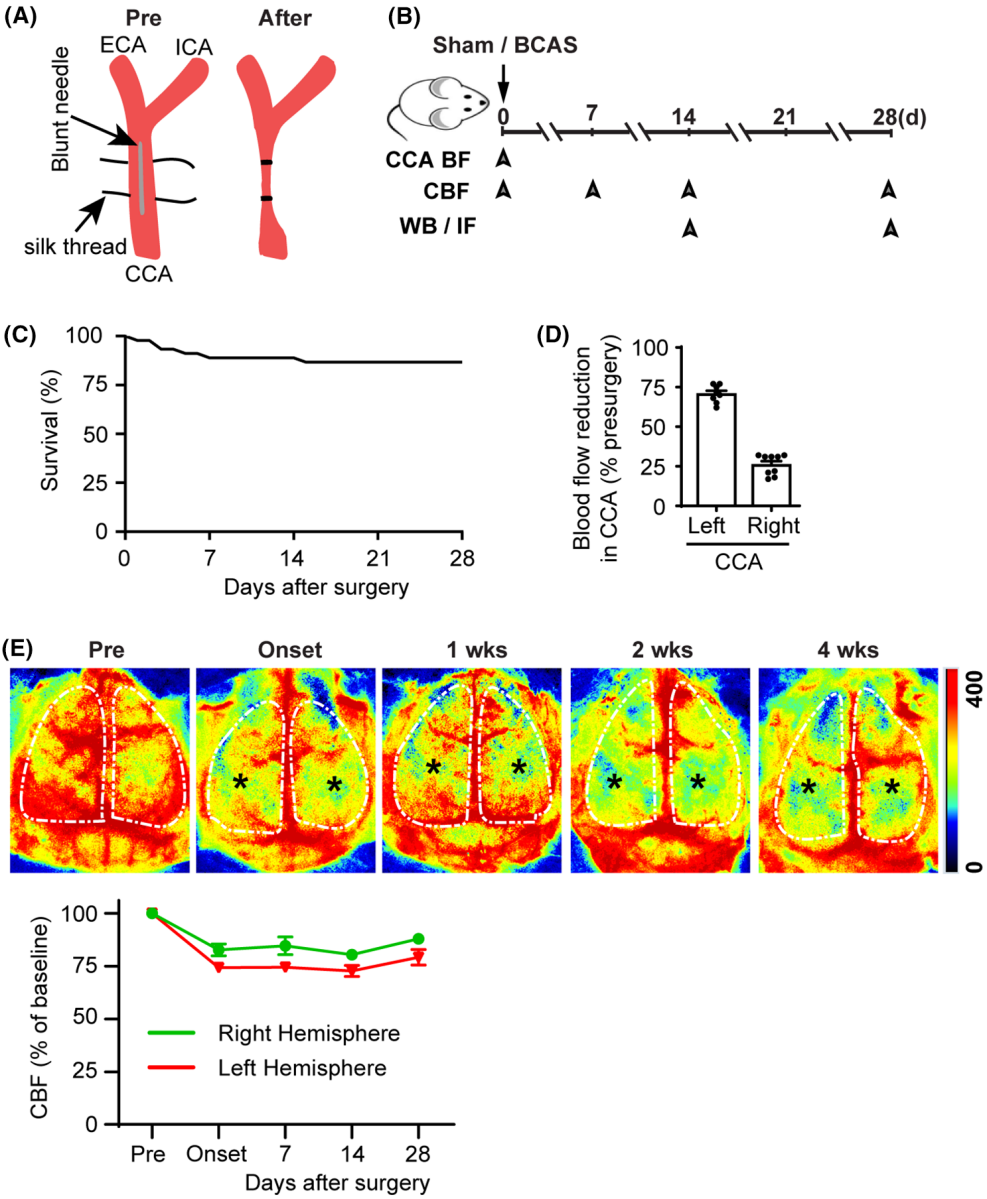
Chronic cerebral hypoperfusion and cognitive impairment in mice following bilateral carotid artery stenosis (BCAS). (A) Illustration of common carotid artery (CCA) stenosis. (B) Experimental protocol and outcome measurements. CCA—common carotid artery; BF—blood flow; CBF—cerebral blood flow; WB—Western blotting; IF—immunofluorescence. (C) Percent survival over 4 weeks after BCAS surgery. (D) Percent blood flow reduction in CCAs after BCAS compared to pre‐surgery baseline. Data are mean ± SEM; *n* = 9. (E) Representative images of cerebral blood flow (CBF) measured by two‐dimensional laser speckle imaging during 0–4 weeks post‐surgery. Black stars indicate brain regions with hypoperfusion. Quantification of CBF (lower panel), white dotted area from upper panel of E used for quantification. Data are mean ± SEM; *n* = 2.

To induce CCH in mice, we employed a suture‐ligated BCAS model with little modification which has been recently established in our laboratory.^17^ C57BL/6J male mice were 10–12 weeks of age (weighing 25–30 g) during the sham or BCAS surgery. Mice were anesthetized with isoflurane (anesthesia was induced with 2% isoflurane in 70/30 N_2_O/O_2_ gas mixture for 3 minutes and maintained during surgery at 1.5%) and placed in supine position. Through a midline incision, both common carotid arteries (CCA) were carefully exposed and isolated from the vagus nerves and surrounding tissues. A blunt 30‐gauge needle (diameter 0.31 mm) was placed on the right CCA (Figure 1A). The artery was doubly ligated with 7–0 silk suture and the needle was gently removed. Similarly, another 33‐gauge needle (diameter 0.21 mm) was placed on the left CCA, the artery was also doubly ligated with 7–0 silk suture, and the needle was gently removed. Sham animals underwent the same procedure but without artery ligation. CCA blood flow before ligation and immediately after needle removal was measured (Figure 1B) using the perivascular flow module (Transonic Systems) with a 0.5 PSB flow probe as described recently.^17^ The probe was sterilized with 70% ethanol and then placed externally to either the left or right CCA to measure the flow. The incision was sutured, analgesia bupivacaine (100 μL 0.25%) was infiltrated topically, and the animal was placed in a cage and monitored for recovery from anesthesia. The body temperature was monitored and maintained at 36.5 ± 0.5°C throughout the experiment by a heating blanket. After recovery, animals were returned to their cages with free access to food and water.

### 2.3 Cerebral blood flow (CBF) measurement

Cerebral blood flow in mice was measured using a two‐dimensional laser speckle contrast analysis system (PeriCam PSI high resolution with PIMSoft, Perimed, Sweden) as described before.^18^ After mice were anesthetized with isoflurane (2.0% in 70% N_2_O and 30% O_2_ for induction, and 1.5% for maintenance), a midline incision was made in the scalp and the exposed skull surface was cleaned with sterile normal saline. Raw speckle images of regions of interest (ROIs) covering the parietal lobe in each hemisphere were taken with a camera placed 10 cm above the skull. Laser speckle images were taken at baseline, 5 min, and 7, 15, and 28 days post‐surgery. Considering that isoflurane may affect CBF in our study,^19^ we maintained administration time and concentration of isoflurane consistent for each animal (i.e., 2% isoflurane in 70/30 N_2_O/O_2_ gas mixture for 3 min in anesthesia induction and 1.5% isoflurane in the same gas mixture for maintenance throughout ~20‐min surgical time) to minimize potential confounding effects of isoflurane. Additionally, body temperature was maintained at 36.5 ± 0.5°C with a heating pad during surgery.

### 2.4 Administration of SPAK inhibitor ZT‐1a

BCAS mice were randomly allocated to receive either vehicle DMSO (2 mL/kg) or ZT‐1a (5 mg/kg/day, every 3 days) via intraperitoneal (i.p.) injection starting from days 14 to 35 post‐BCAS surgery. We selected the ZT‐1a dose and treatment frequency based on our previous study that 1 day of ZT‐1a treatment exhibited efficacy until 7 days post‐stroke.^20^ Sham control mice (which underwent bilateral common carotid artery isolation procedures without artery ligation) received no treatments.

### 2.5 Neurological behavioral function tests

To evaluate learning and memory function in mice, the Morris water maze (MWM) test was conducted in a blinded manner on days 30, 31, 32, 33, and 34 after BCAS surgery. The MWM paradigm is an open‐field procedure in which mice learn to find a hidden platform in order to escape from a forced swimming task. A circular tank was filled with water at 22°C (±1°C) to a depth of 33 cm. A round (10‐cm‐diameter) Plexiglass platform submerged 0.5 cm below water level was left in the same location (north, east, south, or west, 31 cm from the edge) for the duration of each trial. The learning trials were conducted over 5 days, with 4 trials per day. For each of the four trials, mice were placed in the pool facing the wall at one of four starting locations (northeast, southeast, southwest, and northwest) and allowed to swim for a maximum of 90 s. If the mice found the platform, they were allowed to remain on it for 30 s; mice not finding the platform were placed on it and allowed to stay there for 30 s. To assess spatial memory function, a probe trial was conducted on the 6th day of testing without the hidden platform in the pool. Each trial was videotaped via a ceiling‐mounted video camera, and latency and swim speed were calculated. After each session, the mouse was placed in a staging cage with a clean dry towel. Half the cage was warmed with a heating pad to allow the mouse to thermoregulate.

### 2.6 Magnetic resonance imaging (MRI) DTI of ex vivo brain

Following neurocognitive functional tests, mice were anesthetized with 3% isoflurane in 70% N_2_O and 30% O_2_ and transcardially perfused with 4% paraformaldehyde (PFA) and decapitated.^20^ Brains were maintained within the skull to avoid anatomical deformation and fixed in 4% PFA overnight, then stored in PBS solution at 4°C. MRI was performed at 500 MHz using a Bruker AV3HD 11.7 T/89 mm vertical bore small animal MRI scanner, equipped with a 20‐mm quadrature radiofrequency (RF) coil and Paravision 6.0.1 software (Bruker Biospin, Billerica, MA). Following positioning and pilot scans, T2‐weighted images (T2WI) were acquired using a rapid acquisition with relaxation enhancement (RARE) sequence, with the following parameters: echo time/repetition time (TE/TR) = 20/4000 ms, averages = 8, 160 × 160 matrix, 25 slices with a 0.5 mm slice thickness, a RARE factor = 4, and a field of view (FOV) of 16 × 16 mm. Hippocampal atrophy or signal abnormality (low or high signal intensity) was identified as injury on T2‐weighted images by one expert in small animal MRI imaging. A diffusion tensor imaging (DTI) dataset covering the entire brain was collected using a multislice spin‐echo sequence with five reference and 30 noncollinear diffusion‐weighted images with the following parameters: TE/TR = 22/2800 ms, two averages, matrix size = 160 × 160, FOV = 16 × 16 mm, 25 axial slices, slice thickness = 0.5 mm, b‐value = 3000 s/mm^2^, and Δ/δ = 11/5 ms. MRI DTI data were analyzed with DSI Studio (http://dsistudio.labsolver.org/). In a blinded manner, regions of interest (ROIs) were drawn for the corpus callosum (CC), external capsule (EC), and hippocampus. Fractional anisotropy (FA), radial diffusivity (RD), and mean diffusivity (MD) values were determined for each ROI.

### 2.7 Preparation of plasma membrane protein fractions

Brain homogenates were prepared as previously described.^21^ Mice were deeply anesthetized with 4% isoflurane vaporized in N_2_O and O_2_ (3:2), perfused with saline, and decapitated. The contralateral (CL) and ipsilateral (IL) brain tissues were dissected in five volumes of cold homogenization buffer (0.25 M sucrose, 10 mM Tris–HCl, 1 mM EDTA, pH 7.4, and protease and phosphatase inhibitor cocktail [Pierce]). Brain tissues were gently homogenized with a tissue grinder (Kontes, Vineland, NJ, USA) for 10 strokes in homogenization buffer. The homogenized samples were centrifuged at 1000 *g* at 4°C for 10 min. The supernatant was collected and centrifuged at ~200,000 *g* for 30 min using a Beckman OptimaTM XL‐80k ultracentrifuge. The cytosolic fraction (supernatant) and crude membrane pellet were collected. The pellet was resuspended in the homogenization buffer. Protein content in both membrane and cytosolic fractions was determined by the standard bicinchoninic acid method.

### 2.8 Immunoblotting

Protein samples (40 μg) were boiled in sample buffer (Thermo Scientific, Rockford, IL, USA) for 5 min, resolved by 7.5% sodium dodecyl sulfate polyacrylamide‐gel electrophoresis, and electrotransferred onto a polyvinylidene difluoride membrane. The membrane was incubated with appropriate primary antibodies (GFAP, 1:1000; MBP, 1:500; pSPAK/pOSR1, 1:500; pNKCC1, 1:300; tSPAK/OSR1, 1:500; tNKCC1, 1:3000; WNK1, 1:200; WNK2, 1:200; WNK3, 1:200; WNK4, 1:300; and Na‐K ATPase α‐subunit, 1:15,000) at 4°C overnight. The membrane was washed with TBST (Tris‐buffered saline, 0.05% Tween‐20) and incubated with an appropriate secondary antibody conjugated with horseradish peroxidase (anti‐rabbit antibody or anti‐mouse antibody, 1:2000) for 1 h at room temperature. Protein bands were visualized using enhanced chemiluminescence agents. The densities of bands were measured with ImageJ. Expression of Na^+^‐K^+^ pump α1 subunit was used as a loading control for membrane proteins.

### 2.9 Immunofluorescence staining

Immunofluorescent staining for C3d, S100A10, NKCC1, pNKCC1, GFAP, Iba‐1, MBP neurofilament, SMI32, NG‐2, APC, caspase 3, and NeuN were performed as described before.^22^ Mice were deeply anesthetized with 4% isoflurane and transcardially perfused with 0.1 M PBS (pH 7.4), followed by ice‐cold 4% PFA in 0.1 M PBS as described before. Brains were cryoprotected with 30% sucrose after an overnight post‐fixation in 4% PFA. Coronal sections (25 μm, at the level of 1.18 mm anterior to bregma) were selected and processed for immunofluorescent staining. The sections were incubated with blocking solution (10% normal goat serum and 0.15% Triton X‐100 in PBS) for 1 h at room temperature and were then incubated with primary antibodies against C3d (goat, 1:100), MBP (rabbit, 1:400), NF‐200 (rabbit, 1:800), SMI32 (mouse; 1:100), NKCC1 (T4, mouse; 1:100), pNKCC1 (rabbit; 1:100), GFAP (mouse; 1:100), Iba1 (rabbit; 1:200), NeuN (mouse; 1:200), caspase‐3 (rabbit; 1:100), APC (mouse; 1:200), S100A10 (rabbit; 1:200), or Olig‐2 (rabbit; 1:200) in blocking solution at 4°C overnight. After washing in TBS for 3 × 10 min, the sections were incubated with goat anti‐mouse Alexa 488‐conjugated IgG (1:200, Thermo Fisher Scientific), goat anti‐rabbit Alexa 546‐conjugated IgG (1:200, Thermo Fisher Scientific), and donkey anti‐goat Alexa 488‐ conjugated IgG (1:200, Thermo Fisher Scientific) in the blocking solution for 1 h. For negative controls, brain sections were stained with the secondary antibodies only. After washing three times, nuclei were stained with DAPI (1:1000, Thermo Fisher Scientific) for 20 min at 37°C. Sections were mounted with Vectashield mounting medium (Vector Laboratories). Fluorescent images were captured under a 40× lens with 2× magnification using a Nikon A1R or Olympus 1000 inverted confocal laser‐scanning microscope. Identical digital imaging acquisition parameters were used, and images were obtained and analyzed in a blinded manner throughout the study.

### 2.10 Statistical analysis

Animal subjects were randomly assigned to different studies and surgical procedures. All data analyses were performed by investigators blinded to experimental conditions. The number of animals studied was 80% powered to detect 25% changes with α (two‐sided) = 0.05. A total of 66 male mice were used in the study. All mice were included in the study except six mice which died 3–15 days after BCAS surgery. Data were expressed as mean ± SEM. All data were assessed for normal distribution using D'Agostino and Person normality test and Shapiro–Wilk normality test. Data that did not exhibit a normal/Gaussian distribution were analyzed via a nonparametric equivalent. Statistical significance was determined using Student's *t*‐test, or one‐way or two‐way ANOVA, followed by Tukey's post hoc test for multiple comparisons (GraphPad Prism 6.0, San Diego, CA, USA). Escape latency times in MWM test were analyzed by the two‐way repeated‐measures ANOVA. A probability value less than 0.05 was considered statistically significant.

## 3 RESULTS

### 3.1 The BCAS mice displayed chronic cerebral hypoperfusion and memory impairments

Clinically, most patients with intracranial atherosclerotic stenosis suffer from permanent stenosis without complete blockage^23^ and display unilateral and/or asymmetric stenosis.^24^, ^25^ Therefore, to mimic clinical atherosclerotic stenosis with permanent common carotid artery (CCA) hypoperfusion, we employed an asymmetric BCAS model by doubly ligating the CCA, guided with a 30‐gauge needle (0.31 mm diameter) in the right CCA and a 33‐gauge needle (0.21 mm diameter) in the left CCA with a 7–0 silk suture (Figure 1A).^17^ The suture‐ligated BCAS model had a mortality rate of ~13% within 4 weeks post‐surgery (Figure 1C). Compared to Sham‐Ctrl, the BCAS procedure in young adult C57BL/6J mice (male, 10–12 weeks) led to reduction of both CCA blood flow (~70% in the left, and ~25% in the right CCA) at the onset of the ligation surgery (Figure 1D). Moreover, asymmetric BCAS procedure resulted in sustained cerebral blood flow (CBF) reduction in both hemispheres over 0–4 weeks post‐surgery (star mark; Figure 1E). The BCAS mice exhibited significant learning and memory deficits by 4 weeks post‐surgery as assessed by the MWM test (data not shown). The sustained bilateral cerebral hypoperfusion led to lesions in both hemispheres, assessed by myelin basic protein (MBP) loss (data not shown); therefore, we presented averaged data collected from both hemispheres in our studies.

### 3.2 BCAS‐induced upregulation of the NKCC1 protein in GFAP^+^‐reactive astrocytes in white matter

**FIGURE 2:**
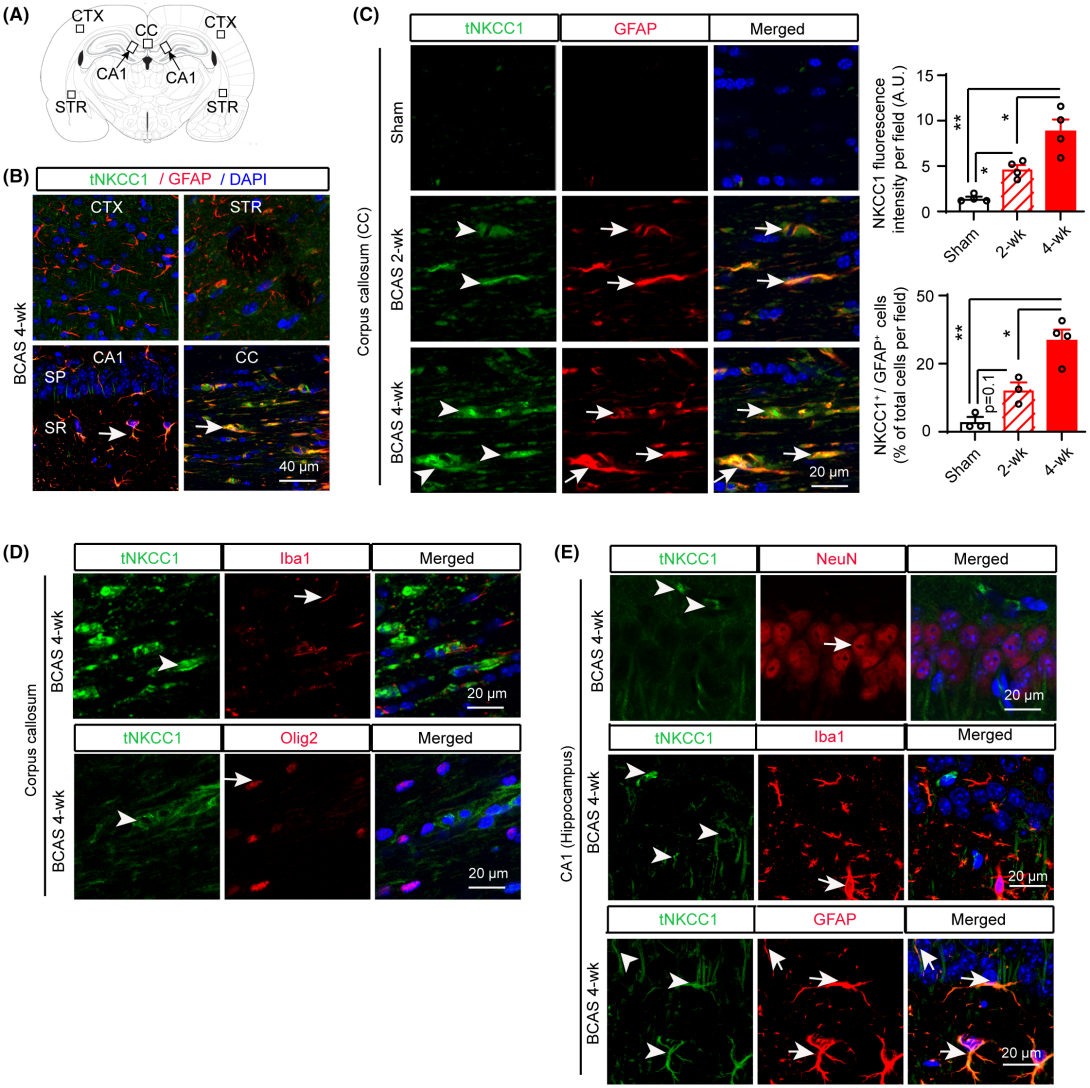
Total NKCC1 protein expression in mouse brains after BCAS. (A) Brain section illustrates sample collection in the cortex (CTX), corpus callosum (CC), hippocampal CA1 subfield (stratum pyramidale [SP] and stratum radiatum [SR]), and striatum (STR) areas. (B) Compared to CTX, STR, and CA1 regions, a robust increase in tNKCC1 protein expression in GFAP^+^ astrocytes (arrow) was detected in the CC at 4 weeks after BCAS surgery. (C) Time‐dependent tNKCC1 protein expression (arrowhead) in GFAP^+^ astrocytes (arrow) at 2 weeks and 4 weeks after BCAS. Data are mean ± SEM; one‐way ANOVA, Tukey's post hoc test; *n* = 4, *p < 0.05, **p < 0.01. (D) In the CC, the increase in tNKCC1 protein expression (arrowhead) does not colocalize with Iba^+^ microglia/macrophages (arrow) or Olig2^+^ oligodendrocytes at 4 weeks after BCAS. (E) In the hippocampal CA1, an increase in tNKCC1 protein expression (arrowhead) does not colocalize with NeuN^+^ neurons (arrow) or Iba1^+^ microglia/ macrophages (arrow) but colocalizes with GFAP^+^ astrocytes (arrow) at 4 weeks after BCAS.

We examined changes in total NKCC1 (tNKCC1) protein expression in different brain regions including the cortex (CTX), striatum (STR), hippocampal CA1, and corpus callosum (CC), at 2 and 4 weeks post‐surgery (Figure 2A). Among these regions, the CC showed the most robust increase in tNKCC1 protein expression in GFAP^+^‐reactive astrocytes following BCAS at 4 weeks post‐surgery (Figure 2B). Therefore, we focused our investigation on the association between NKCC1 activity and white matter‐reactive astrogliosis as well as WML in the CC and external capsule (EC) tracts. In Figure 2C, ShamCtrl mice displayed barely detectable levels of tNKCC1 protein expression in the CC. However, by 2 weeks post‐BCAS surgery, tNKCC1 immunofluorescence intensity in the CC tract was increased by ~3 folds (middle panel, *p* < 0.05), and further elevated to ~6 folds by 4 weeks (arrowhead, lower panel, *p* < 0.05). Notably, the upregulated tNKCC1 protein was located along the fiber tract and particularly enriched within GFAP^+^‐reactive astrocytes (arrow). The upregulation of tNKCC1 and GFAP protein expression in the CC exhibited a significant correlation, with a Pearson's correlation coefficient of *r* = 0.84 (*p* < 0.001) (Figure S1). These results suggested that the BCAS induced the expression of NKCC1 in white matter GFAP^+^ astrocytes. We also investigated whether BCAS induced NKCC1 expression in microglia/macrophages and oligodendrocytes within the CC region. However, we did not find any colocalization of NKCC1 with microglia/macrophages marker Iba1 or the oligodendrocytes marker Olig2 (Figure 2D). In the hippocampal CA1 region, we observed a modest increase in tNKCC1 protein expression, which was mostly colocalized with GFAP^+^ astrocytes but not with NeuN^+^ neurons or Iba1^+^ microglia/macrophages (Figure 2E). Collectively these findings confirm that BCAS upregulates tNKCC1 protein expression in GFAP^+^‐reactive astrocytes in the white matter and some in the hippocampal region. Therefore, in the following experiments, we investigated whether pathological stimulation of NKCC1 protein in white matter astrocytes contributes to the transformation of cytotoxic/homeostatic reactive astrocytes, OL death, and demyelination following BCAS.

### 3.3 Detection of BCAS‐induced WNK‐SPAK‐NKCC1‐signaling activation via immunoblotting

**FIGURE 3:**
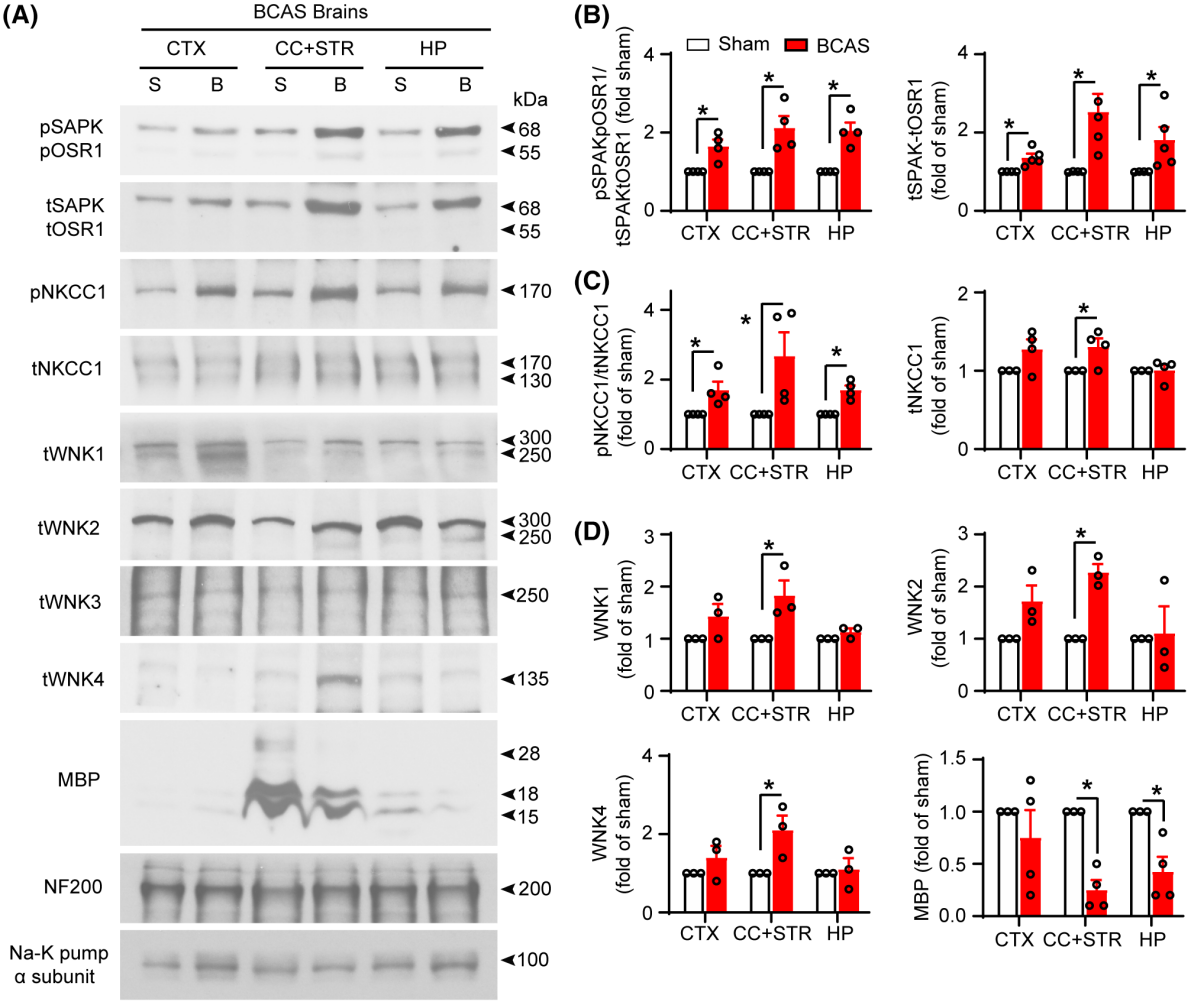
Stimulation of WNK‐SPAK‐NKCC1 complex in BCAS mouse brains. (A) Representative immunoblots of increased WNK‐SPAK/OSR1‐NKCC1 complex proteins and decreased myelin basic proteins (MBP) in BCAS‐induced hypoperfused mouse brains (membrane protein fractions) at 4 weeks after surgery. Na‐K pump (α subunit) was used as a loading control. (B–D) Quantitative analyses. Data are mean ± SEM; Student's *t* test; *n* = 3–4, *p < 0.05. B, BCAS; BCAS, bilateral carotid artery stenosis; CC, corpus callosum; CTX, cortex; HP, hippocampus; S, sham; STR, striatum.

WNK‐SPAK‐NKCC1 signaling plays a critical role in numerous neurological disorders including ischemic stroke, TBI, and post‐hemorrhagic hydrocephalus.^21^, ^26^, ^27^ In this study, we quantified changes in the WNK‐SPAK‐NKCC1‐signaling proteins in different brain regions including CTX, CC + STR, and HP, of both sham and BCAS mice by western blotting. Compared to the Sham‐Ctrl, BCAS triggered significant increases in the expression of pSPAK/pOSR1 (pSer383/pSer325) and pNKCC1 (pThr212) at 4 weeks post‐BCAS (*p* < 0.05, Figure 3A–C). Moreover, total SPAK/OSR1, tWNK1, tWNK2, and tWNK4 were significantly upregulated (*p* < 0.05, Figure 3B–D). Similar to total NKCC1 expression, we observed increased pNKCC1 expression in GFAP^+^ astrocytes, but not in NeuN^+^ neurons (CA1 region of the hippocampus) or Iba1^+^ microglia/macrophages (CA1 region of the hippocampus and the CC tract; Figure S2). On the other hand, BCAS resulted in a massive reduction in MBP expression in the CC, while expression of neurofilament heavy‐chain NF200 remained unchanged throughout the tested samples (Figure 3A,D), suggesting demyelination is the main cause of WML observed in our BCAS model but not the consequence of axonal degeneration and loss. These data further confirm that the activation of the WNK‐SPAK‐NKCC1‐signaling pathway and demyelination coincide in mouse brains, especially, in white matter regions, following BCAS.

### 3.4 Post‐BCAS administration of the SPAK inhibitor ZT‐1a rescued memory impairments

**FIGURE 4:**
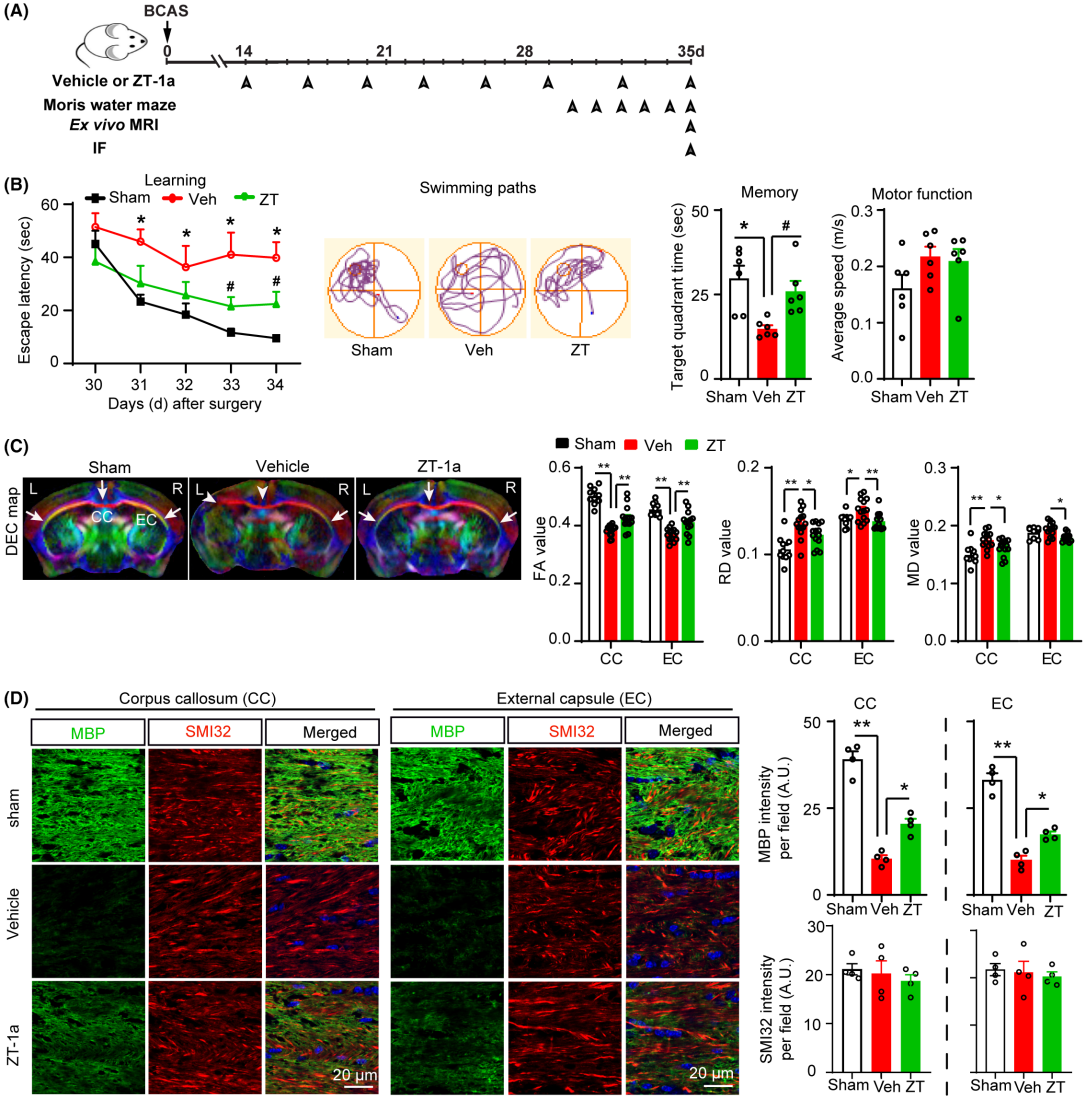
SPAK inhibitor ZT‐1a prevents memory impairments and protects white matter microstructure integrity and MBP loss after BCAS. (A) Post‐BCAS administration of vehicle (Veh) or SPAK inhibitor ZT‐1a (5 mg/kg, i.p., during 14–35 days after BCAS, every 3 days), Morris water maze test, ex vivo MRI, and immunofluorescence (IF). (B) Compared to Veh‐treated BCAS mice, ZT‐a treatment reduces the escape latency times and swimming path, and increases target quadrant time. Data are mean ± SEM; two‐way repeated‐measures ANOVA, Tukey's post hoc test; *n* = 6, *p < 0.05 versus sham, ^#^ *p* < 0.05 versus Veh. (C) Representative images of DEC maps of ex vivo brain from sham, vehicle (Veh)‐treated, and ZT‐1a (ZT)‐treated mice at 5 weeks post‐BCAS detected by MRI‐DTI. Bar graphs show quantitative analyses of fractional anisotropy (FA), radial diffusivity (RD), and mean diffusivity (MD) of CC and EC. Data are mean ± SEM; one‐way ANOVA, Tukey's post hoc test; *n* = 7 (data points represent values from both hemispheres), *p < 0.05, **p < 0.01. (D) Changes in myelin basic protein (MBP) and nonphosphorylated neurofilament heavy chain (SMI32) in CC and EC of brains in B–C. Quantitative analyses of MBP and SMI32 immunofluorescence. Data are mean ± SEM; one‐way ANOVA, Tukey's post hoc test; *n* = 3–4, *p < 0.05, **p < 0.01.

We then tested the efficacy of a newly developed specific SPAK inhibitor ZT‐1a on improving cognitive functions and white matter integrity in BCAS mice. We administered ZT‐1a when there was already a significant WML observed, specifically 14 days after BCAS surgery (Figure S3). Either vehicle DMSO (Veh, 2 mL/kg) or ZT‐1a (5 mg/kg, every 72 h, i.p.) was administered and mice were sacrificed at 35 days post‐BCAS (Figure 4A). Compared to Sham‐Ctrl, the Veh‐treated BCAS mice demonstrated spatial learning impairments (no significant reduction in escape latency during 5‐day MWM test). In contrast, ZT‐1a‐treated BCAS mice displayed significantly reduced escape latency (Figure 4B). Additionally, in the spatial probe trial, ZT‐1a‐treated mice spent significantly longer time in the target quadrant (26.3 ± 4.3 s) than the Veh‐treated BCAS mice (15.3 ± 1.2 s, *p* < 0.05), indicating better retention of spatial memory function (Figure 4B). Motor functions were similar in all groups, as evidenced by similar average swimming speeds in the Sham, Veh, and ZT‐1a‐treated mice. These results suggest that the SPAK inhibitor ZT‐1a rescued BCAS‐induced learning and memory impairments.

### 3.5 The SPAK inhibitor ZT‐1a treatment in BCAS mice preserved white matter integrity

Next, we examined whether improvement of cognitive deficits in the ZT‐1a‐treated BCAS mice is due to protection of white matter microstructure. Following the completion of neurological and cognitive tests, we harvested ex vivo brains for DTI MRI analysis of WML and analyzed the FA, RD, and MD of the CC and EC tracts. Representative DEC map images in Figure 4C demonstrated severe loss of axonal fibers (arrowheads) in the CC and left EC of Veh‐treated BCAS brains. In contrast, the ZT‐1a‐treated mice exhibited better preservation of CC and EC tract integrity in both hemispheres (arrows). These observations are consistent with changes in FA, RD, and MD values (right panels). The Veh‐treated BCAS group exhibited significantly reduced FA values in the CC tract (0.383 ± 0.004) compared to the Sham‐Ctrl group (0.502 ± 0.009, *p* < 0.01). This was corroborated by increased RD (0.135 ± 0.004 vs. 0.106 ± 0.004) and MD (0.174 ± 0.001 vs. 0.149 ± 0.005) values in the Veh group. In contrast, ZT‐1a‐treated BCAS mice displayed significantly higher FA values and lower RD and MD values than the Veh‐treated group (*p* < 0.05), reflecting preserved white matter microstructure. We then measured MBP and SMI32 expression levels in these brains by immunofluorescence staining. Figure 4D revealed a massive loss of MBP protein expression in the CC and EC tracts of Veh‐treated BCAS brains compared to sham control group, which is consistent with the immunoblotting findings where MBP was robustly decreased in the white matter region (CC + STR) of BCAS brains compared to sham control (Figure 3A). Conversely, the ZT‐1a‐treated brains exhibited significantly less MBP loss (*p* < 0.05, Figure 4D). Although we observed massive MBP loss, SMI32 (a marker of nonphosphorylated pathological neurofilament heavy chain) levels were not significantly changed in the CC and EC tract of Veh‐treated BCAS mice compared to sham mice, which is consistent with immunoblotting results in Figure 3A. Collectively, these findings suggest that ZT‐1a prevents BCAS‐induced myelin loss and preserves white matter integrity.

### 3.6 ZT‐1a prevented BCAS‐mediated loss of OPCs and OL in CC and EC

**FIGURE 5:**
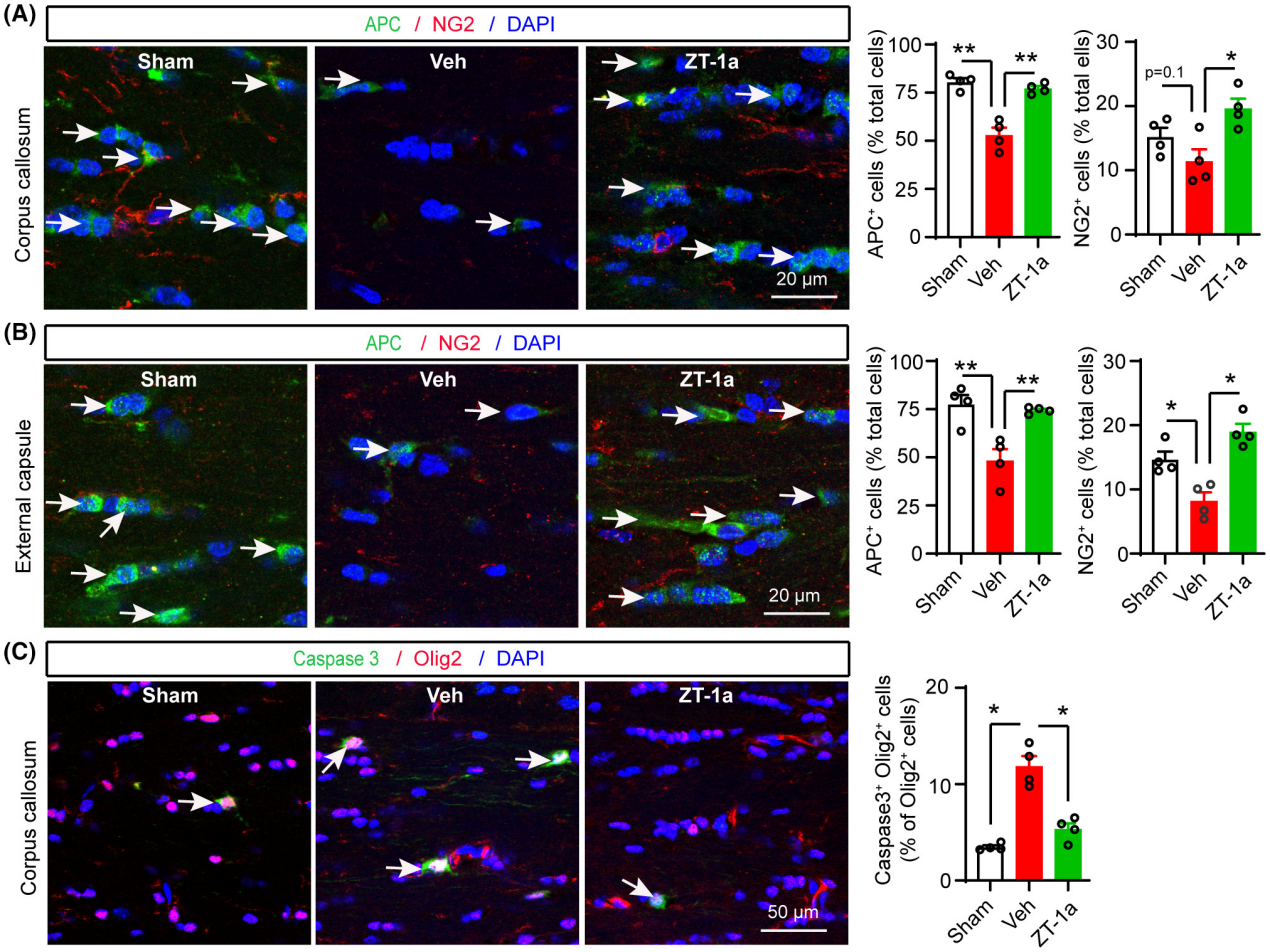
SPAK inhibitor ZT‐1a prevents BCAS‐induced loss of both OPCs and OL in CC and EC. (A–C) Double‐immunofluorescence analysis of APC/NG2 in CC and EC, and caspase 3^+^/Olig2^+^ cells in CC of sham, Veh‐, and ZT‐1a‐treated brains at 5 weeks after BCAS surgery. The same cohort as Figure 4. Nuclei were counterstained with DAPI. Data are mean ± SEM; one‐way ANOVA, Tukey's post hoc test; *n* = 4, *p < 0.05, **p < 0.01.

Next, we investigated whether better preserved white matter microstructure displayed in the ZT‐1a‐treated mice was due to increased oligodendrogenesis and/or decreased OL death. To test this mechanism, we performed immunofluorescence analysis on the same cohort of brains to assess the counts of oligodendrocyte progenitor cell (OPC and NG2^+^), differentiated mature OL (APC^+^), and apoptotic OL (caspase 3^+^ Olig2^+^) in sham, Veh‐treated, and ZT‐1a‐treated BCAS mice. As illustrated in Figure 5A–C, compared to sham mice, the Veh‐treated BCAS mice exhibited significant loss of APC^+^ mature OL counts and NG2^+^ OPC cell counts in the CC and EC tracts, along with an increase in caspase 3^+^ OL counts in the CC at 5 weeks post‐BCAS. In contrast, the ZT‐1a‐treated BCAS mice demonstrated significantly higher counts of APC^+^ cells and NG2^+^ cells in the CC and EC tracts, as well as lower counts of caspase 3^+^ OL in the CC, compared to the Veh‐treated group (Figure 5A–C). These findings suggest that ZT‐1a treatment prevented OPC and OL death and enhanced the differentiation and maturation of OPC into mature OL.

### 3.7 ZT‐1a attenuated BCAS‐induced increased expression of tNKCC1, pNKCC1, and GFAP in white matter astrocytes

**FIGURE 6:**
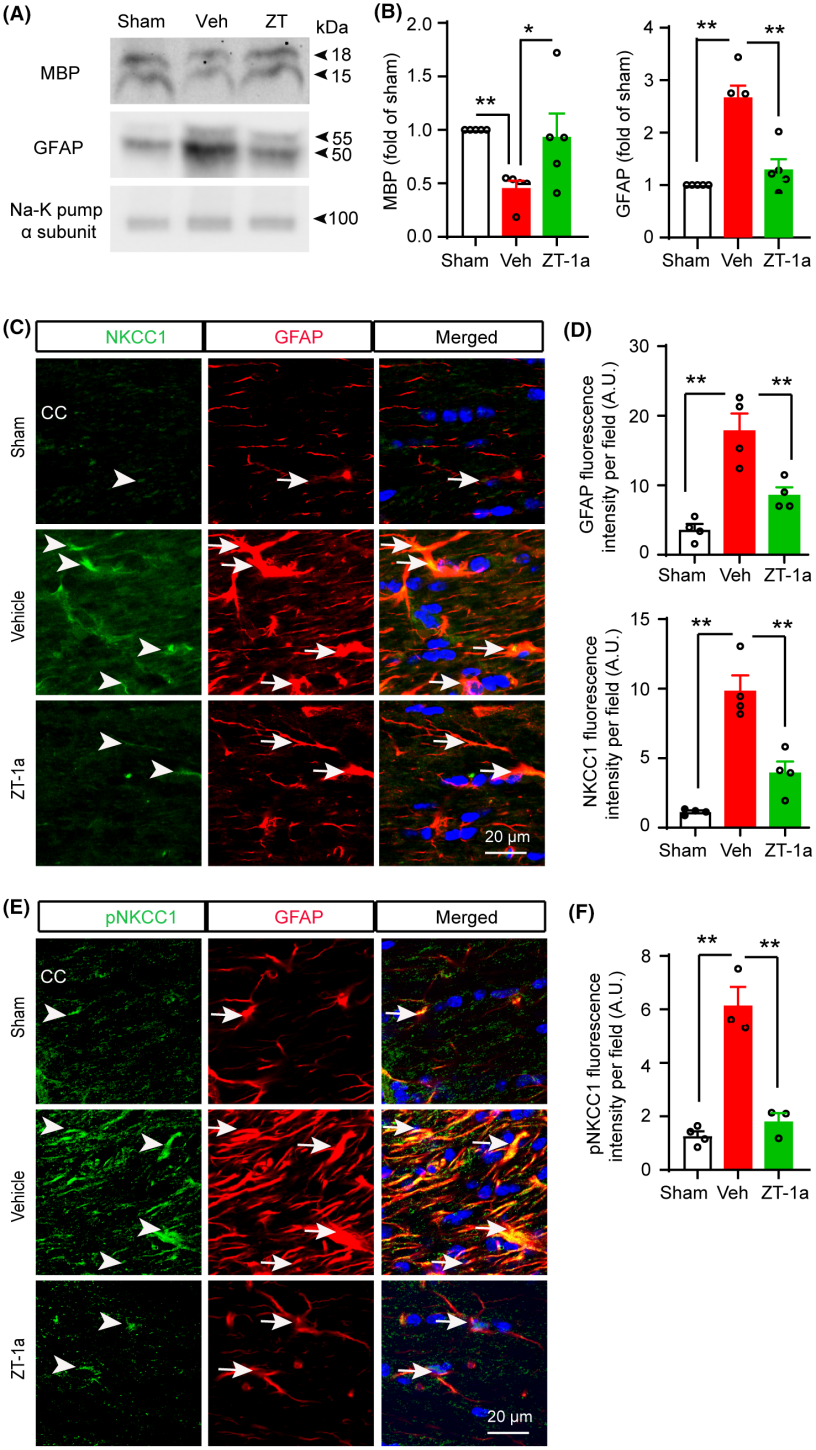
SPAK inhibitor ZT‐1a inhibits expressions of tNKCC1, pNKCC1, and GFAP, and prevents MBP loss in the corpus callosum (CC) after BCAS. (A, B) ZT‐1a treatment prevents BCAS‐induced increased expression of GFAP and loss of MBP in mouse brain 5 weeks after BCAS. Data are mean ± SEM; one‐way ANOVA, Tukey's post hoc test; *n* = 3–4; *p < 0.05, **p < 0.01. (C–F) ZT‐1a‐treated mice exhibit decreased expressions of NKCC1 and GFAP proteins in GFAP^+^ astrocytes in CC compared to Veh‐treated mice at 5 weeks after BCAS. Data are mean ± SEM; one‐way ANOVA, Tukey's post hoc test; *n* = 3–4; **p < 0.01.

Western blot analysis of the vehicle‐treated and ZT‐1a‐treated BCAS brain tissues revealed that ZT‐1a treatment prevented MBP loss in parallel with a decrease in GFAP expression in BCAS mouse brains (Figure 6A,B), suggesting a relationship between reactive astrogliosis and OL death. Since we hypothesized that SPAK‐NKCC1 complex activity is involved in reactive astrogliosis, we used a pharmacological inhibitor ZT‐1a to block SPAK‐NKCC1 activity and examined the effects of ZT‐1a on attenuating tNKCC1, pNKCC1, and GFAP expression in mouse BCAS brains. Immunofluorescence analysis using validated primary antibodies for tNKCC1 and pNKCC1 (Figure S4) demonstrated a profound increase in the expression and phosphorylation of NKCC1 in GFAP^+^ astrocytes (*p* < 0.01, Figure 6C–F) in the Veh‐treated BCAS group compared to the sham group. In contrast, ZT‐1a treatment markedly reduced the expression and phosphorylation of NKCC1 in GFAP^+^ astrocytes of BCAS brains (arrow, Figure 6C–F), further supporting our hypothesis that increased NKCC1 activity is involved in reactive astrogliosis, which contributes to OL death and demyelination.

### 3.8 ZT‐1a attenuated BCAS‐induced cytotoxic reactive astrocytes and increased homeostatic astrocytes in white matter tracts of mouse brains

**FIGURE 7:**
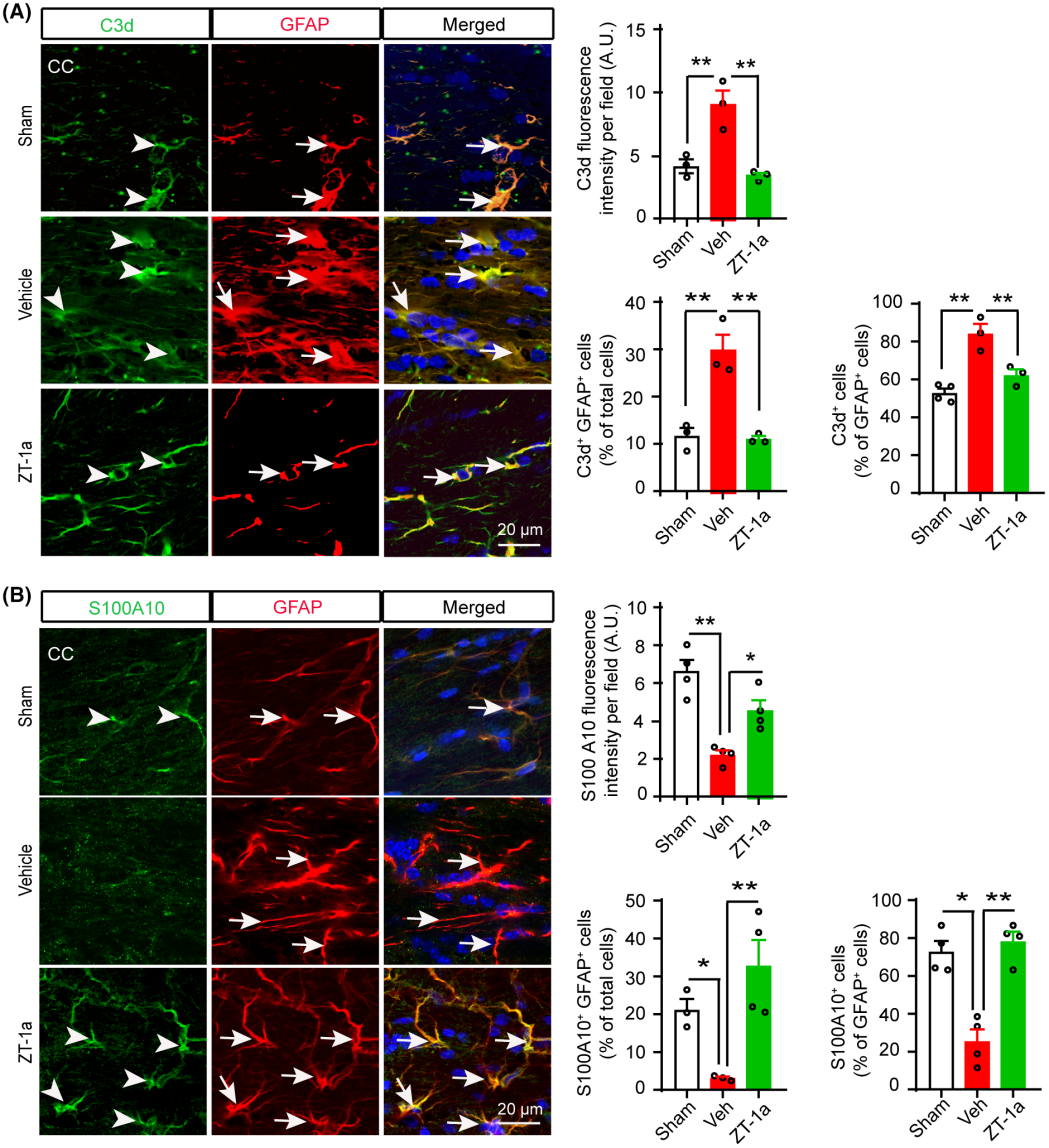
SPAK‐NKCC1 complex inhibition attenuates increment of cytotoxic astrocytes and increases homeostatic astrocytes in the BCAS mouse brains. (A) ZT‐1a‐treated mice exhibit decreased expressions of C3d protein in GFAP^+^ astrocytes in CC compared to Veh‐treated mice at 5 weeks after BCAS. Bar graphs show quantitative analyses of C3d fluorescence intensity, C3d^+^GFAP^+^ cells (% of total cells), and C3d^+^ cells (% of GFAP^+^ cells). Data are mean ± SEM; one‐way ANOVA, Tukey's post hoc test; *n* = 3–4; *p < 0.05, **p < 0.01. (B) ZT‐1a treatment increases S100A10^+^ homeostatic astrocytes in CC compared to vehicle‐treated mice at 5 weeks after BCAS. Bar graphs represent quantitative analyses of S100A10 fluorescence intensity, S100A10^+^GFAP^+^ cells (% of total cells), and S100A10^+^ cells (% of GFAP^+^ cells). Data are mean ± SEM; one‐way ANOVA, Tukey's post hoc test; *n* = 3–4; *p < 0.05, **p < 0.01.

Although reactive astrogliosis is the hallmark of brain injury and degenerative diseases including VCID,^22^, ^28^ the precise role of reactive astrocytes in brain cell death or CNS repair is not clear. Recent studies have identified two distinct types of reactive astrocytes based on their gene expression profile and functions: (1) proinflammatory cytotoxic astrocytes and (2) homeostatic protective astrocytes.^29^, ^30^ Therefore, we examined the effects of SPAK inhibitor ZT‐1a on the transformation of the cytotoxic/homeostatic reactive astrocytes in the mouse brain following BCAS by immunofluorescence analysis with specific antibodies against C3d and S100A10 (Figure 7 and Figure S5), which are markers for cytotoxic and homeostatic reactive astrocytes, respectively.^31^ We observed a significant increase in C3d expression and the number of C3d^+^ cells as well as a significant decrease in S100A10 immunofluorescence intensity and the number of S100A10^+^ cells in both the CC and EC regions of Veh‐treated mouse brains compared to sham mice. Interestingly, in ZT‐1a‐treated mice, there was a significant reduction in C3d expression and the number of C3d^+^ cells, accompanied by significant increase in S100A10 immunofluorescence intensity and the number of S100A10^+^ cells, in both the CC and EC region compared to Veh‐treated mouse brains. These results suggest that the selective inhibition of SPAK‐NKCC1 activity by ZT‐1a prevents the transformation of quiescent astrocytes into cytotoxic reactive astrocytes and increases the number of homeostatic astrocytes in the mouse brain following BCAS.

## 4 DISCUSSION

Chronic cerebral hypoperfusion causes OL death and demyelination which is the major cause of cognitive impairment and dementia.^32^, ^33^ Among various VCID models, the BCAS model induces CCH and recapitulates key characteristics of VCID, including astrogliosis, inflammation, WML, and cognitive impairments.^34^ BCAS triggers reactive astrogliosis in white matter,^31^, ^35^ cortex, striatum, and hippocampus.^36^ Reactive astrogliosis triggered by injury or ischemia can initiate a feed‐forward inflammatory process and contribute to disease progression. Although the functions of reactive astrocytes have been a subject of debate, growing evidence suggests that C3d^+^‐reactive astrocytes are toxic to cortical neurons, embryonic spinal motor neurons, and mature, differentiated oligodendrocytes in cell culture and mouse brain.^29^, ^30^ However, the mechanism by which reactive astrocytes induce cell death in brain cells remains unclear. In this study, we observed reactive astrogliosis in corpus callosum, external capsule, and striatum regions at 2–5 weeks after BCAS surgery. Importantly, an upregulation of NKCC1 protein was detected in white matter tracts which were colocalized with GFAP^+^‐reactive astrocytes. Moreover, increased phosphorylated and total SPAK protein, and total WNK1/2/4 protein were concurrently detected in white matter (striatum and CC) tissues in the BCAS mouse brains. Therefore, we speculated that CCH‐mediated activation of WNK‐SPAK‐NKCC1 cascade contributes to reactive astrogliosis, inflammation, and subsequent demyelination and cognitive impairment, and inhibition of SPAK‐NKCC1 complex would prevent reactive astrogliosis, demyelination, and cognitive impairment.

Reactive astrocytes are also able to slow and impair the differentiation of OPC into OL in vitro and in vivo^29^, ^31^ and induce caspase‐3‐dependent apoptotic cell death in neurons and OL.^29^ Similarly, in this study, we detected a substantial increase in C3d^+^GFAP^+^ cytotoxic astrocytes in white matter tracts (CC and EC) at 35 days post‐BCAS coinciding with caspase‐3‐dependent OL death and demyelination. Most importantly, SPAK inhibitor ZT‐1a attenuated transformation of quiescent astrocytes into cytotoxic reactive astrocytes and increased the number of S100A10^+^GFAP^+^ homeostatic astrocytes in the white matter tracts of mouse brains following BCAS. These results support the notion that C3d^+^‐reactive astrocytes play a detrimental role in BCAS‐induced demyelination, possibly by killing APC^+^ mature OL and NG^+^ OPC and inhibiting differentiation and maturation of OPC into OL. Indeed, a recent study showed an association between impaired OL maturation and increased C3d^+^ cytotoxic reactive astrocytes in a mouse BCAS model,^31^ further supporting our hypothesis. However, the specific toxic molecule(s) secreted by cytotoxic reactive astrocytes that induce OPC/OL death and inhibit OPC proliferation and differentiation are yet to be identified.^29^ Interestingly, a recent study reported that the leukemia inhibitory factor (LIF) secreted from GFAP^+^ astrocytes plays a protective role against BCAS‐induced memory impairment in WT C57BL/6J mice during early stage of VCID.^37^ Kakae et al. (2023) showed that astrocytic LIF enhanced differentiation of myelinating OL at 2 weeks after BCAS but not at 4 weeks after BCAS.^37^ In our study, we found increased myelinating OL in ZT‐1a‐treated BCAS mice at 4 weeks after BCAS. Whether ZT‐1a treatment affects astrocyte LIF secretion warrants further study.

Systemic administration of the specific SPAK inhibitor, ZT‐1a, in mice significantly reduced NKCC1 protein expression and phosphorylation in white matter astrocytes, resulting in attenuated gliosis, demyelination, and improved cognitive function in BCAS mice. Our results are consistent with a prior study^38^ in which inhibiting NKCC1 with bumetanide attenuated CCH‐induced WML and cognitive impairment, suggesting the SPAK‐NKCC1 complex as a potential therapeutic target for VCID. Although we observed a robust efficacy of ZT‐1a during the early stage of VCID (2–5 weeks post‐BCAS), we did not investigate BCAS‐induced pathophysiological changes at delayed time points, or did we assess the efficacy of ZT‐1a at advanced stages. Therefore, further study is required to assess the effects of ZT‐1a during the advanced stage of VCID and to establish the optimal doses of ZT‐1a.

It is important to note that SPAK and NKCC1 proteins are widely expressed in the CNS both in glia and neurons,^39^ and play an important role in the synaptic transmission, blood–cerebrospinal fluid barrier integrity, inflammation, as well as brain injury.^40^, ^41^, ^42^ BCAS‐induced memory impairment is not only associated with WML but also hippocampal neurodegeneration.^43^ However, in this needle‐gauged BCAS model, we did not observe a significant neuronal death in the hippocampus at 5 weeks after BCAS (Figure S6), suggesting that BCAS‐induced memory impairment observed in our study was not associated with hippocampal neurodegeneration, at least, at the early stage of VCID (4–5 weeks post‐BCAS). Our results are consistent with previous reports that BCAS‐induced hippocampal neurodegeneration is a delayed phenomenon.^44^, ^45^ However, we cannot exclude any potential effect of ZT‐1a on other types of brain cells, including microglia and endothelial cells, which are integral components of the neurovascular unit and can influence astrocytic swelling and ischemic injury.^46^ Taken together, better drug‐like properties of ZT‐1a including specificity for SPAK kinase, CNS permeability,^20^ and robust efficacy in mouse BCAS model reveal the therapeutic potential of ZT‐1a for the treatment of VCID.

NKCC1 phosphorylation and activity are regulated by upstream WNK and SPAK/OSR1 kinases. In this study, we observed significant upregulation of total WNK (1, 2, and 4), SPAK, and NKCC1 protein expression in mostly white matter and HP at 4 weeks after BCAS. Upregulation of tNKCC1 protein primarily localized within GFAP^+^‐reactive astrocytes in the white matter tracts. However, the underlying mechanisms of how WNK‐SPAK‐NKCC1 cascade proteins are upregulated in astrocytes remain unknown. In our previous study,^21^ through bioinformatics and ChIP‐qPCR assays, we identified that human and mouse *Wnk1/2/4*, *Spak*, and *Nkcc1* gene promoters contain multiple NF‐κB‐binding sites (5′‐GGGRNYYYCC‐3′). We also found that ischemia‐induced NF‐κB activity regulates transcriptional upregulation of the WNK‐SPAK‐NKCC1 complex genes in ischemic stroke mouse brains.^21^ Therefore, it is plausible that the upregulation of WNK‐SPAK‐NKCC1 cascade proteins in BCAS mouse brains is a result of increased NF‐κB recruitment on *Wnk1*, *Wnk2*, *Wnk4*, *Spak*, and *Nkcc1* gene promoters which remains to be elucidated in future studies. In fact, NF‐κB activation has been found to be involved in BCAS‐induced astrogliosis and selective inhibition of astrocytic NF‐κB activity in *Gfap‐IkBα/dn* mice (expressing a dominant negative inhibitor of NF‐κB) attenuated white matter astrogliosis after BCAS^47^ and supports our hypothesis.

Consistent with our previous study,^21^ we found that ZT‐1a treatment not only reduced BCAS‐induced phosphorylation of NKCC1 protein but also decreased the expression of total NKCC1 protein in the current study. Whether ZT‐1a affects *Nkcc1* transcription or NKCC1 protein translation is not known. A likely mechanism could be that ZT‐1a reduced brain inflammation which in turn resulted in reduced NK‐κB‐mediated *Nkcc1* transcription. SPAK kinase regulates immune responses in many inflammatory disorders including ischemia/reperfusion injury,^48^ inflammatory bowel disease,^49^ and post‐hemorrhagic hydrocephalus.^27^ Using multi‐omic approach, recently Robert et al.^50^ demonstrated that SPAK kinase acts as both transducer of pro‐inflammatory cytokine toll‐like receptor 4 (TLR4) signals and regulators of multiple‐ion transporters including NKCC1 in neuroinflammatory disorders. Therefore, it is possible that inhibition of SPAK activity by ZT‐1a checks TLR4/NF‐κB inflammatory cascade, potentially contributing to the reduced expression of *Nkcc1* in mouse brain.

## 5 CONCLUSION

In this study, we present evidence that BCAS‐induced CCH stimulates the WNK‐SPAK‐NKCC1 cascade in the brain, which is, at least in part, responsible for reactive astrogliosis, OL death, WML, and memory impairments. Most importantly, post‐BCAS administration of ZT‐1a in mice dampens reactive astrogliosis, OL death, and WML and improves learning and memory function. These findings revealed the potential of pharmacological blockade of the SPAK‐NKCC1 complex in reducing CCH‐induced WML and cognitive impairment and underscored the therapeutic potential of ZT‐1a in ameliorating the detrimental effects of cytotoxic reactive astrocytes in VCID and other neurodegenerative diseases.

## AUTHOR CONTRIBUTIONS

Study conception and design: M.I.H.B. and D.S.; experiment or data collection: M.I.H.B., K.H., M.T.S., F.C., I.J., Z.W., M.S.R., and L.M.F.; data analysis: M.I.H.B., K.H., F.C., L.M.F., and T.K.H.; data interpretation: M.I.H.B., D.S., L.M.F., T.K.H., and G.C.; writing – manuscript preparation and intellectual input: M.I.H.B., D.S., K.H., M.T.S., R.I., L.M.F., T.K.H., S.W.C., X.D., and G.C.; supervision and administration: M.I.H.B. All authors approved the final version of the manuscript.

## FUNDING INFORMATION

Research reported in this publication was supported by the National Institute Of Neurological Disorders and Stroke (NINDS) of the National Institutes of Health under Award Number R01NS119166 (M.I.H.B.) and RF1NS117509 (G.C.), and Veterans Affairs grants VA BX003923 (G.C.) and I01BX002891‐01A1 (D.S.). We also thank the Border Biological Research Center (BBRC) Core Facilities at The University of Texas at El Paso (UTEP) for their support, which was funded by NIH grant (5G12M007590).

## CONFLICT OF INTEREST STATEMENT

The authors declare that they have no competing interests.

## Supporting information


Appendix S1.

Appendix S2.

## Data Availability

All data generated or analyzed during this study are included in this published article and its supplementary information files or are available on reasonable request from the corresponding author.
