# Supplementary material for: SPAK inhibitor ZT‐1a attenuates reactive astrogliosis and oligodendrocyte degeneration in a mouse model of vascular dementia
Source: CNS Neurosci Ther. 2024 Mar 3;30(3):e14654. doi: 10.1111/cns.14654 (PMC10909630; doi:10.1111/cns.14654)
Supplement: Supplementary file 1 — Appendix S1. [file CNS-30-e14654-s002.pdf]

Supplemental Information

SPAK inhibitor ZT-1a attenuates reactive astrogliosis and oligodendrocyte degeneration in a mouse model of vascular dementia

Mohammad Iqbal H. Bhuiyan[1,2,3,4], Khadija Habib[1], Md Tipu Sultan[1], Fenghua Chen[2], Israt Jahan[1,2], Zhongfang Weng[2], Md Shamim Rahman[1], Rabia Islam[5], Lesley M. Foley[6], T. Kevin Hitchens[6,7], Xianming Deng[8], Scott W. Canna[9], Dandan Sun[2,3,4], Guodong Cao[2,4]

[1]Department of Pharmaceutical Sciences, School of Pharmacy, University of Texas at El Paso, El Paso, TX, 79902, USA
[2]Department of Neurology, University of Pittsburgh, Pittsburgh, Pennsylvania, 15213, USA
[3]Pittsburgh Institute for Neurodegenerative Disorders, University of Pittsburgh, Pittsburgh, Pennsylvania, 15213, USA
[4]Veterans Affairs Pittsburgh Health Care System Pittsburgh Healthcare System, Geriatric Research Education and Clinical Center, Pittsburgh, Pennsylvania, 15240, USA
[5]Nostrum Hospital, Dhaka, 1230, Bangladesh
[6]Animal Imaging Center, University of Pittsburgh, Pittsburgh, Pennsylvania, 15213, USA
[7]Department of Neurobiology, University of Pittsburgh, Pittsburgh, Pennsylvania, 15213, USA
[8]State Key Laboratory of Cellular Stress Biology, School of Life Sciences, Xiamen University, Xiamen, Fujian, China
[9]Department of Pediatric Rheumatology, The Children's Hospital of Philadelphia, Philadelphia, PA 19104, USA

Supplementary materials

The protein sample buffer and bicinchoninic acid assay reagent were purchased from Thermo-Fisher Scientific (Rockford, IL). 5-chloro-N-(5-chloro-4-((4-chlorophenyl)(cyano)methyl)-2-methylphenyl)-2-hydroxybenzamide (“ZT-1a”) was developed in our laboratory (Ref: Nat, Comm).DAPI (4,6-diamino-2-phenylindole, dihydrochloride) was obtained from Life Technologies Corporation (Carlsbad, CA). Mouse/rat complement component anti-C3d antibody (AF2655) from R&D Systems, Inc. (Minneapolis, USA). Anti-MBP (ab40390) and anti-WNK2 (ab192397) antibodies were from Abcam plc (Cambridge, UK). Rabbit Anti-NG2 chondroitin sulfate proteoglycan antibody (AB5320) from Sigma-Aldrich Inc. (ST. LOUIS MO, USA). Mouse anti-GFAP (3670s) and rabbit anti-caspase-3 (14220) antibodies were from Cell Signaling Technology (Danvers, MA). Rabbit anti-neurofilament 200 (N4142) and mouse anti-NeuN antibodies (MAB377) were from Millipore Corporation (Billerica, MA). Goat anti-Iba1 (NB100-1028) antibody was purchased from Novus Biologicals (Centennial, CO). Anti-phospho-NKCC1 (pNKCC1; T206), pSPAK/pOSR1, and total SPAK/OSR1 (tSPAK/tOSR1) antibodies were developed and kindly gifted by Dr. Yang (Taiwan National University) [1-3]. Mouse anti-total NKCC1 monoclonal antibody and mouse anti-Na^+-K^+-ATPase were collected from Developmental Studies Hybridoma Bank (Iowa City, IA). Anti-WNK1 (A301-515-M) from Bethyl Laboratories (Montgomery, TX) [3, 4], anti-WNK3 (WNK31-A) from Alpha Diagnostic (San Antonio, TX) [3], and anti-WNK4 (NB600-284) from Novus Biochemical (Littleton, CO) were purchased. Mouse anti-Neurofilament H (NF-H) (SMI32) were purchased from Biolegend (San Diego, CA #801701]), and rabbit anti-phospho-NKCC1 antibody (Thr212/Thr217) were from Millipore Sigma (Darmstadt, Germany). Horseradish peroxidase (HRP)-conjugated anti-rabbit Ig was from Molecular Probes (Eugene, OR). HRP-conjugated anti-mouse Ig and anti-sheep Ig antibodies, and enhanced chemiluminescence (ECL) reagents were purchased from Pierce (Rockford, IL). Goat-anti-rabbit Alexa Fluor 488 (A11008) and Alexa Fluor 546 (A11035), or goat-anti-mouse Alexa Fluor 546 (A11030), were from Life Technologies (Grand Island, NY). Donkey anti-goat 488 (A-11055) and Donkey anti-rabbit 546 (A10040) were obtained from Thermo-Fisher Scientific.

Supplementary figures:

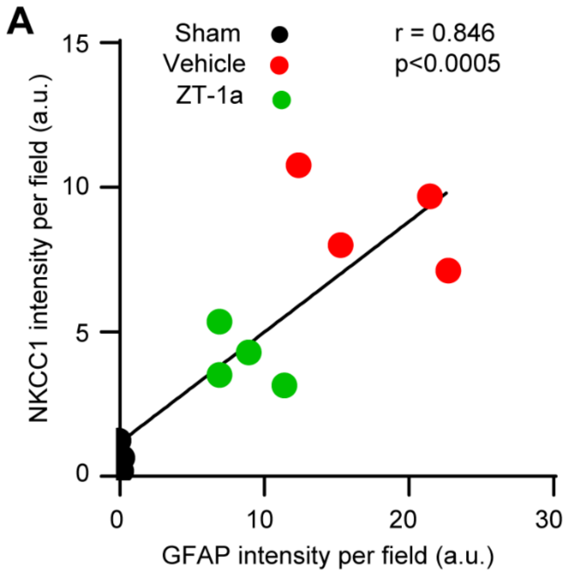

Supplemental Fig. S1. Correlation between brain NKCC1 activation and concurrent GFAP protein expressions in BCAS mice. A Pearson's correlation analysis between brain NKCC1 activation and GFAP expression in BCAS mice from Fig. 6.

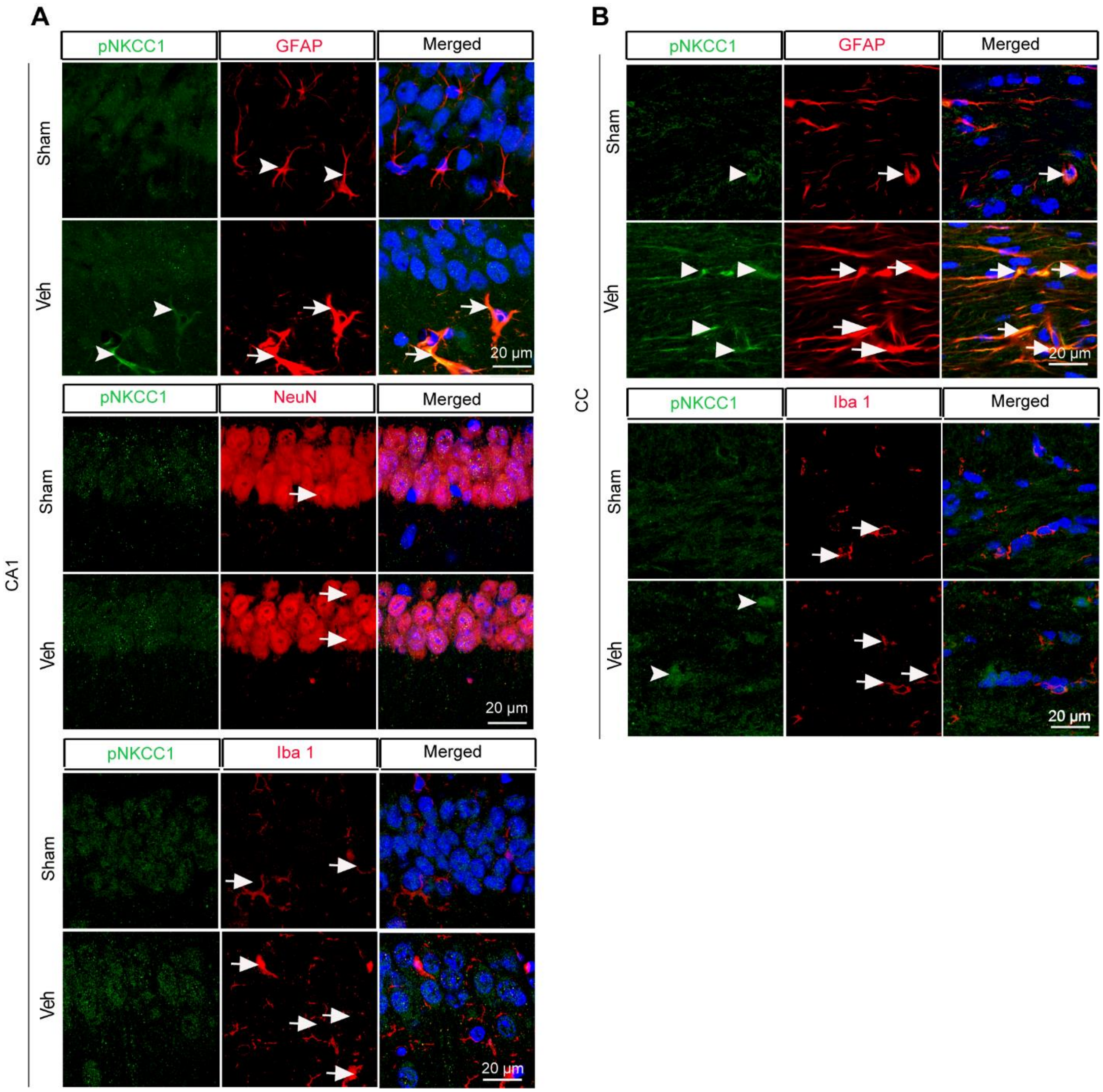

Supplemental Fig. S2. pNKCC1 expression increases in GFAP⁺ astrocytes in the mouse brain after 4-wk BCAS surgery. A Representative pNKCC1/GFAP, pNKCC1/NeuN, and pNKCC1/Iba1 staining of sham and 4-wk mouse brain (hippocampus CA1 region) after BCAS. Nuclei were counterstained with DAPI**. B** Representative pNKCC1/GFAP and pNKCC1/Iba1 staining of sham and 4-wk mouse brain (CC) after BCAS. Nuclei were counterstained with DAPI.

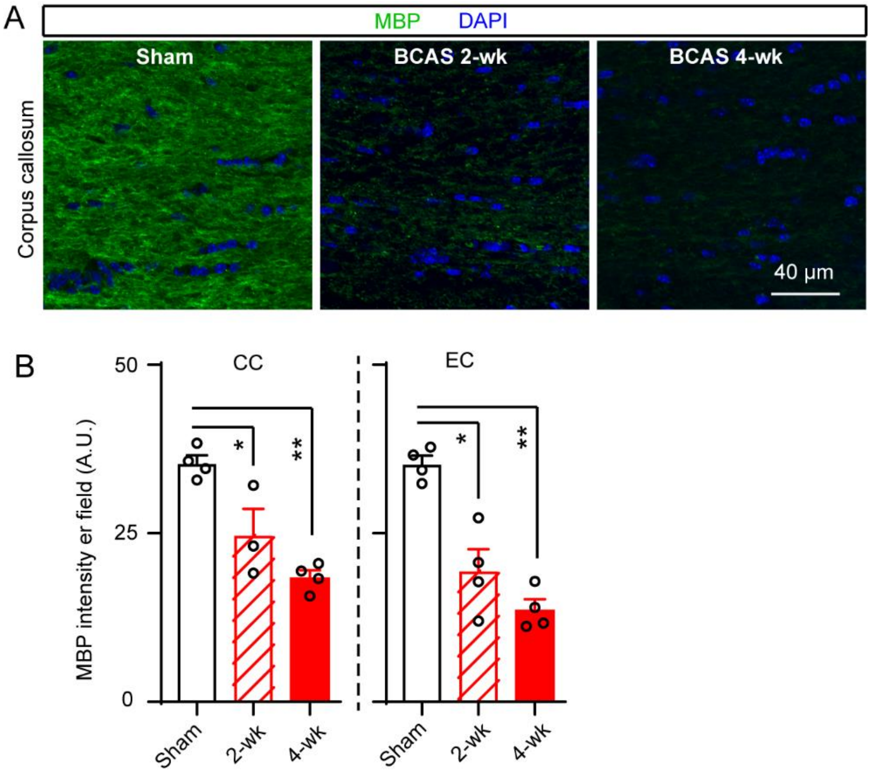

Supplemental Fig. S3. Demyelination in mouse brain following BCAS. A Representative MBP staining of sham, 2-wk, and 4-wk mouse brains after BCAS. Nuclei were counterstained with DAPI. **B** Quantification of MBP intensity. Data are mean ± SEM; one-way ANOVA, Tukey's post-hoc test; n= 4, *P<0.05, **p<0.01.

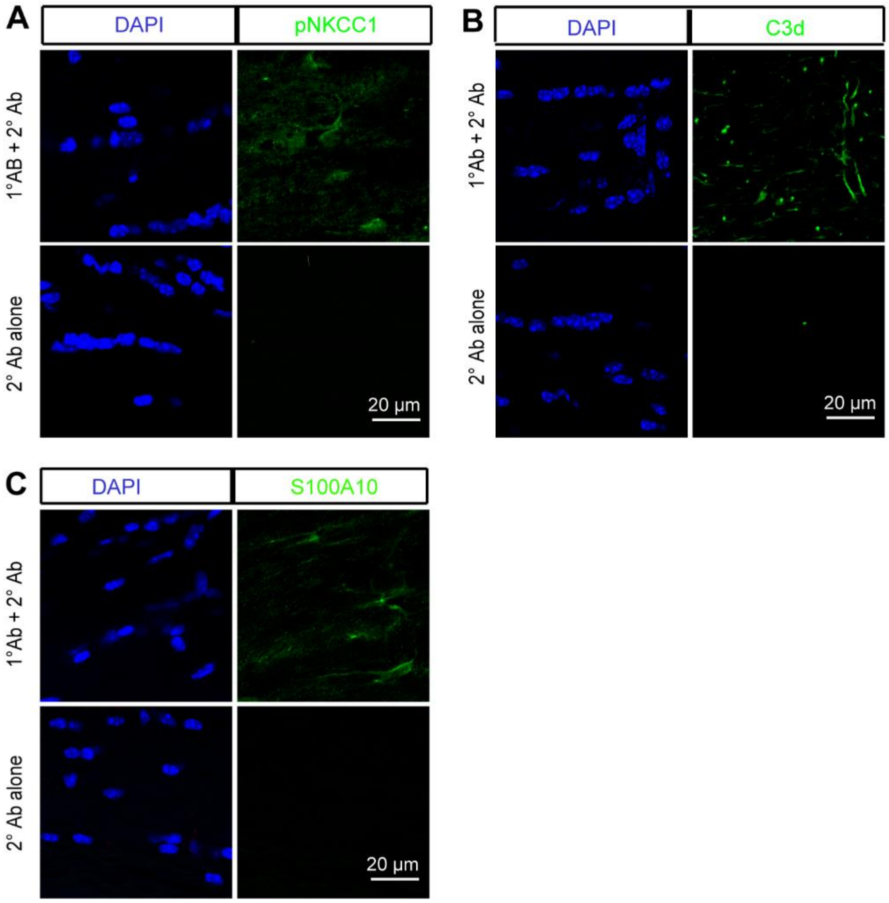

Supplemental Fig. S4. Detection of specific immunofluorescence signals with commercial anti-pNKCC1, anti-C3d, and anti-S100A10 primary antibodies in mouse brain following BCAS. **A** Sham brain sections were stained either with pNKCC1 and respective secondary antibody (upper panel) or with only anti-rabbit secondary antibody conjugated with Alexa Flour 488 (down panel). **B** Sham brain sections were stained either with C3d primary and respective secondary antibodies (upper panel) or with only anti-goat secondary antibody conjugated with Alexa Flour 488 (down panel). Nuclei were counterstained with DAPI. **C** Sham brain sections were stained either with S100A10 primary and respective secondary antibodies (upper panel) or with only anti-rabbit secondary antibody conjugated with Alexa Flour 488 (down panel).

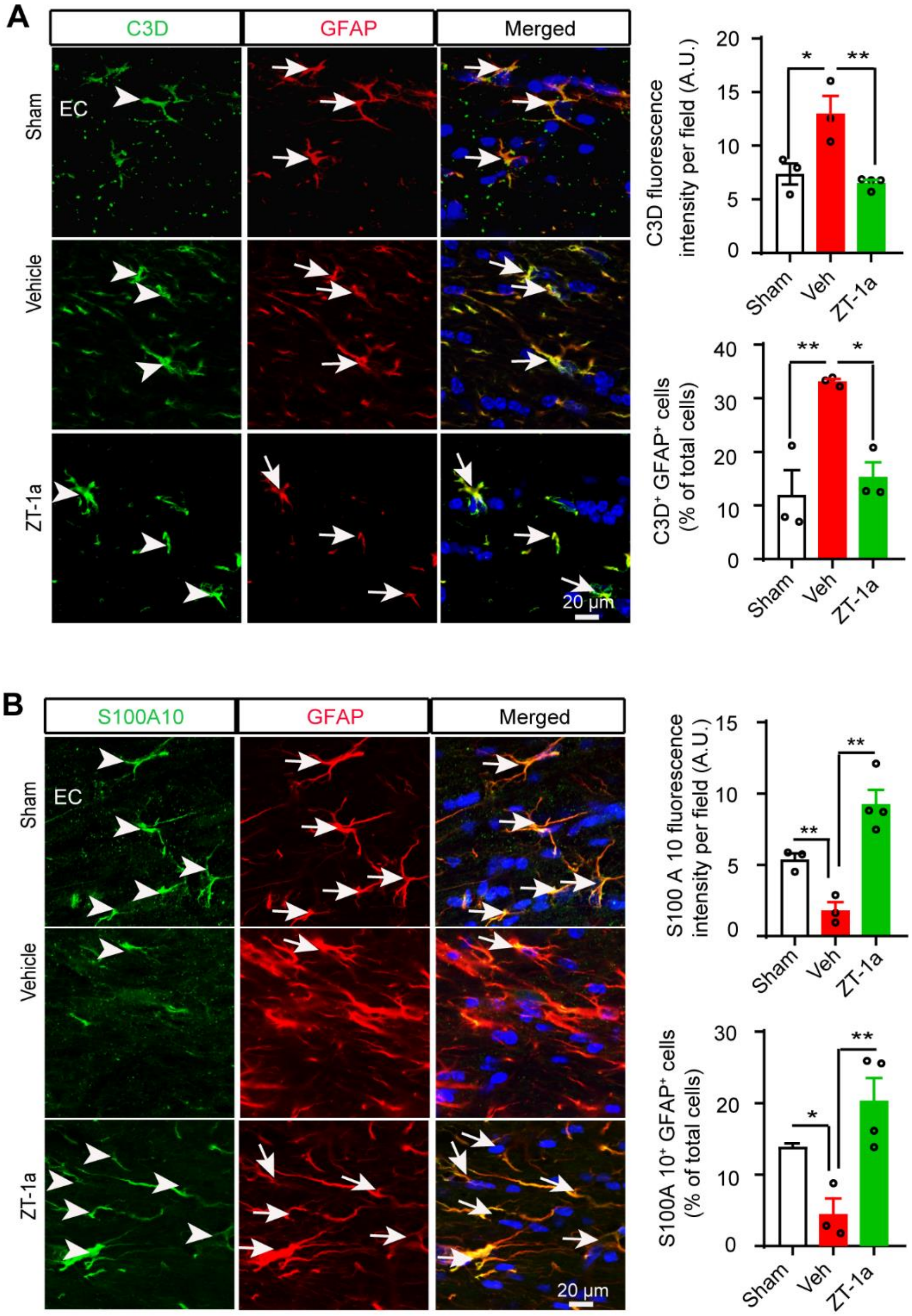

Supplemental Fig. S5. SPAK-NKCC1 complex inhibition abrogates C3d positive reactive astrocytes increments and prevents S100A10 positive astrocytes loss in the BCAS model. A Compared to vehicle-treated mice, ZT-1a treatment decreases the expression of neurotoxic reactive astrocytes in the EC region of the mice brain after BCAS. Bar graphs show quantitative analyses of C3d intensity and $C3d^{+}GFAP^{+}$ cells (% of total cells). Data are mean±SEM; one-way ANOVA, Tukey's post-hoc test; n= 3-4; *p<0.05, **p<0.01. **B** ZT-1a administration protects BCAS-induced homeostatic astrocyte death in the EC compared to vehicle-treated mice at 4 weeks

after BCAS. Bar graphs represent quantitative analyses of S100A10 intensity and S100A10$^+$GFAP$^+$ cells (% of total cells). Data are mean±SEM; one-way ANOVA, Tukey's post-hoc test; $n = 3$-4, $^*p<0.05$ and $^{**}p<0.01$.

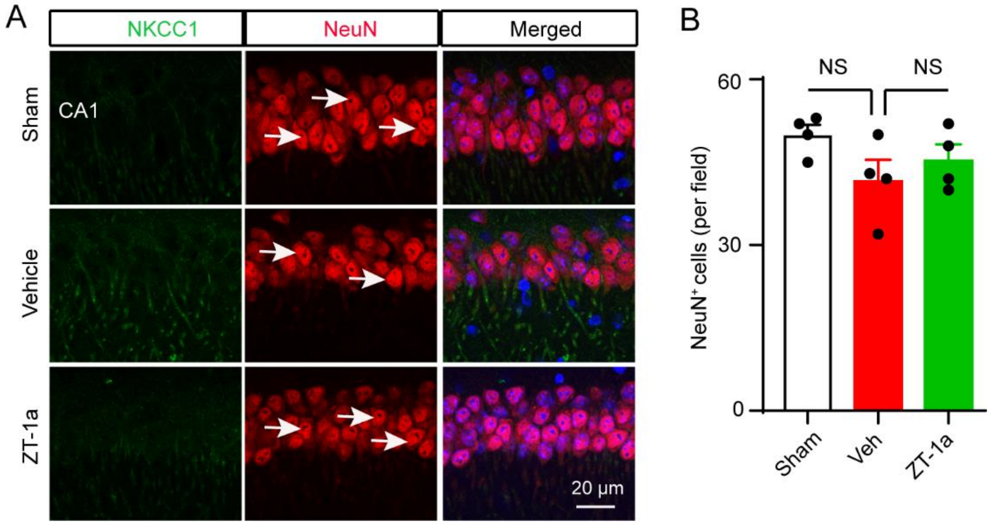

Supplemental Fig. S6. No significant hippocampal CA1 neuronal death 5 weeks after BCAS. **A** Representative image of NKCC1$^+$NeuN$^+$ cells in Sham, Veh-, and ZT-1a-treated groups in the CA1 region of mouse hippocampus at 5 weeks after BCAS. **B** Quantitative analysis of NeuN$^+$ cells. Data are mean ± SEM. n= 4, NS – not significant.

Supplemental References

1. Moriguchi, T., et al., *WNK1 regulates phosphorylation of cation-chloride-coupled cotransporters via the STE20-related kinases, SPAK and OSR1.* J Biol Chem, 2005. **280**(52): p. 42685-93.
2. Yang, S.S., et al., *SPAK-knockout mice manifest Gitelman syndrome and impaired vasoconstriction.* J Am Soc Nephrol, 2010. **21**(11): p. 1868-77.
3. Bhuiyan, M.I.H., et al., *NF-kappaB Signaling-Mediated Activation of WNK-SPAK-NKCC1 Cascade in Worsened Stroke Outcomes of Ang II-Hypertensive Mice.* Stroke, 2022. **53**(5): p. 1720-1734.
4. Furusho, T., et al., *Renal TNFalpha activates the WNK phosphorylation cascade and contributes to salt-sensitive hypertension in chronic kidney disease.* Kidney Int, 2020. **97**(4): p. 713-727.
