# Supplementary figures and images for: SPAK inhibitor ZT‐1a attenuates reactive astrogliosis and oligodendrocyte degeneration in a mouse model of vascular dementia
Source: CNS Neurosci Ther. 2024 Mar 3;30(3):e14654. doi: 10.1111/cns.14654 (PMC10909630; doi:10.1111/cns.14654)

Full gel blots of Fig. 3

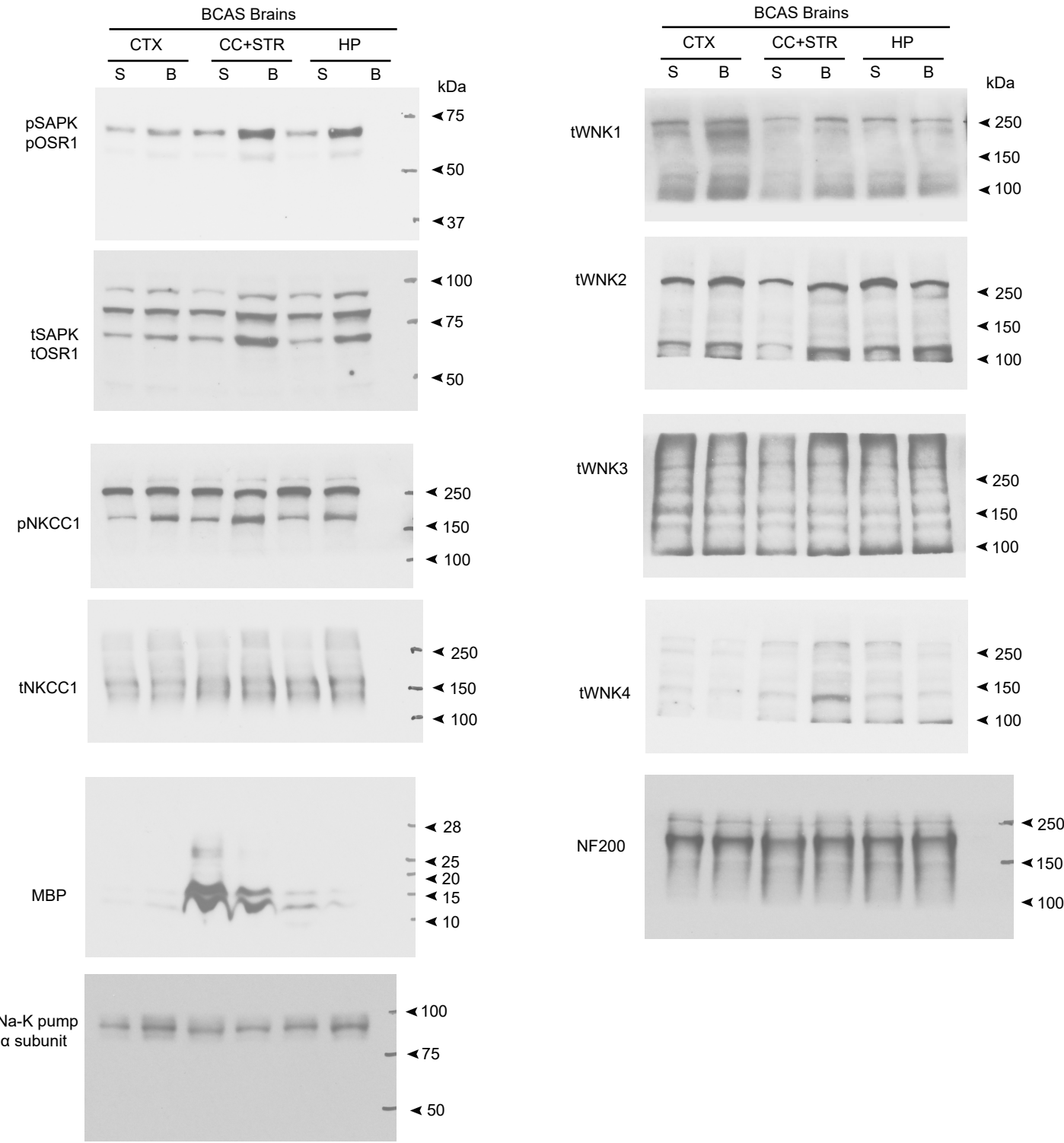

Full gel blots of Fig. 6

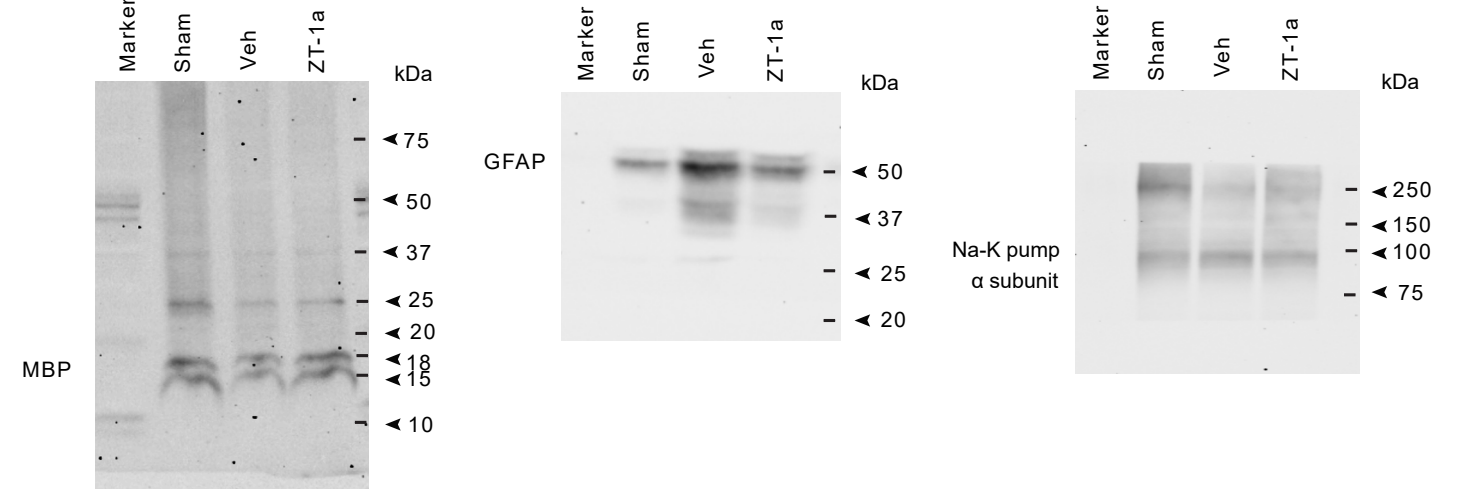

Supplement: Supplementary file 2 — Appendix S2. [file CNS-30-e14654-s001.pdf]
